# Cognitive Remediation Interventions in Autism Spectrum Condition: A Systematic Review

**DOI:** 10.3389/fpsyt.2020.00722

**Published:** 2020-07-24

**Authors:** Yasemin Dandil, Katherine Smith, Emma Kinnaird, Cindy Toloza, Kate Tchanturia

**Affiliations:** ^1^Department of Psychological Medicine, Institute of Psychology, Psychiatry and Neuroscience, King’s College London, London, United Kingdom; ^2^Department of Psychology, National Eating Disorder Service, South London and Maudsley NHS Foundation Trust, London, United Kingdom; ^3^Department of Psychology, Illia State University, Tbilisi, Georgia

**Keywords:** autism, cognitive remediation, interventions, therapy, cognitive training, social cognition, adolescents, adults

## Abstract

**Background:**

Autism spectrum condition (ASC) is a lifelong neurodevelopmental condition characterized by difficulties in social cognition and heterogeneity of executive function which are suggested to be underpinned by neurobiological, prenatal and genetic factors. Cognitive remediation (CR) interventions are frequently used to address cognitive characteristics and improve cognitive and general functioning. However, the evidence is limited for ASC. This systematic review is the first to provide a narrative synthesis of all studies of CR interventions and ASC. The review aimed to delineate the development of research in this area in both adolescents and adults, with implications for clinical practice and future research.

**Method:**

The review was conducted according to the Preferred Reporting Items for Systematic Reviews and Meta‐analysis (PRISMA) statement. The literature was reviewed using the PubMed, PsycINFO, Web of Science, Scopus and Embase from inception to 1st April 2020. Out of 1,503 publications, a total of 13 papers were identified as being relevant for the review.

**Results:**

The 13 studies meeting the inclusion criteria were: four randomized control trials (RCTs); two non-randomized control trials, four case series, two feasibility studies and one case study. A narrative synthesis of the data suggested that CR interventions are potentially effective in improving social cognition and cognitive functioning in ASC. RCTs supported the efficacy of CR interventions in improving social cognition and executive functioning. Non-randomized control trials provided evidence for the effectiveness of social cognition remediation interventions in ASC. Case series and a case study have also supported the feasibility of CR interventions, including reflections on their adaptation for ASC populations and the positive feedback from participants.

**Conclusions:**

CR interventions are potentially effective in improving social cognition and cognitive functioning in ASC. However, the generalizability of the included empirical studies was hampered by several methodological limitations. To further strengthen understandings of the effectiveness of CR interventions for ASC, future RCTs are needed with larger sample sizes in exploring the long-term effectiveness of CR interventions, using age-appropriate valid and reliable outcome measures. They should also consider the heterogeneity in neuropsychological functioning in ASC and the mediating and moderating mechanisms of the CR intervention for ASC.

## Introduction

Autism spectrum disorder (ASD) is a lifelong neurodevelopmental condition characterized by differences in reciprocal social interaction, communication, language, and restricted and repetitive behaviors (1). Although the term ASD is the term used for medical diagnosis, we instead use the term Autistic Spectrum Condition (ASC), which is the preferred term by people with the life experience of this diagnosis (2). In the 1980s, ASC was considered uncommon with a prevalence estimate of around 22 in 10,000 (3). Nonetheless, with increasing recognition the ASC prevalence estimate is now considered to be approximately one in 100 and relatively common (4).

ASC is associated with a spectrum of manifestations and behaviors that affect individuals in different ways. However, these surface differences are theorized to be underpinned by a common cognitive profile: inflexibility of thinking and heightened attention to detail (5, 6). Furthermore, ASC is characterized by difficulties in social cognition and heterogeneity of executive function which are suggested to be underpinned by neurobiological, prenatal and genetic factors (7). A recent systematic review and meta-analysis aimed to systematically map non-social and social cognitive functioning in autistic adults (8). Overall, the findings from 75 included studies comprising of 3,361 autistic people supported the notion that autistic adults show difficulties in non-social and social cognitive domains (theory of mind, verbal learning and memory, emotional processing and perception, and processing speed). This review highlights the significance of identifying remediation interventions that target non-social and social cognition.

The timely identification of ASC can improve quality of life by identifying needs and providing appropriate interventions (9). It has been suggested that cognitive remediation (CR) interventions targeting this underlying cognitive profile may be particularly beneficial for this population (10). CR interventions are an umbrella term for psychological interventions that use cognitive training exercises to remedy difficulties in social cognition and neuropsychological functioning. CR interventions aim to encourage patients to reflect on their thinking styles, planning strategies to enable them to make behavioral changes (11).

The use of cognitive training and later CR remedial interventions were originally developed for use in brain lesions (12, 13) and were then broadened for patients with schizophrenia (14, 15); and adapted further for patients with anorexia nervosa (AN); (11). CR interventions have demonstrated significant improvements in neuropsychological tests of set-shifting (16, 17).

To date, four randomized control trials (RCTs) have been conducted on the use of CR interventions and ASC. The findings from these trials indicate that CR interventions can be effective in improving cognitive flexibility, social cognition and heterogeneity of executive function (10, 18–20). However, the translation of the empirical study findings to further clinical work in this field is hampered by methodological limitations such as heterogeneity in participants clinical presentations, variability in outcome measures used and small sample sizes. Additionally, it is possible that standard CR interventions developed for other populations may benefit from adaptations for ASC populations, an area of research relevant to the successful clinical implementation of these interventions in this field.

Therefore, this systematic review aims to contribute to the field of ASC interventions by presenting a narrative synthesise of all peer-reviewed published studies of CR approaches in this area. To our knowledge, this is the first paper to review the literature specifically for CR interventions and ASC. Hence, the paper aims to add to the field by providing a chronological outline of the current literature and to discuss the implications of these findings for future work in this area.

## Method

### Review and Search Strategy

The review was conducted according to the Preferred Reporting Items for Systematic Reviews and Meta‐analysis (PRISMA) statement (21). The literature was reviewed using the PubMed, PsycINFO, Web of Science, Scopus and Embase from inception to 1st April 2020. The search terms used were autis*—Asperger* and cognit* with remed*—or train*–neuropsychology—cognitive remediation therap*. These terms allowed for multiple spellings, plurals and combinations.

### Inclusion Criteria

The pre-determined inclusion criteria were [1] only published, peer-reviewed English language journals; [2] studies of any design (quantitative or qualitative) focusing on CR interventions and ASC [3] empirical studies that include either adolescents or adults [4] studies using any modality of treatment e.g. individual sessions or group sessions.

The definition of CR interventions used for this review was not restricted to cognitive remediation therapy (CRT) but included other CR approaches such as cognitive enhancement therapy, social cognition remediation programs, brain training tasks, flexibility training, or CR interventions that had been adapted for other populations and used for those with ASC. Additionally, the review included studies examining CR approaches in relation to autistic traits, as well as in populations with diagnosed ASC. Furthermore, no limitations were set regarding age or measure of neuropsychological characteristics. This allowed for a wider search.

### Exclusion Criteria

Non-English language publications (22) and empirical studies that were not published in peer-reviewed journals were excluded. Secondary systematic or narrative reviews on the wider topic of CR interventions in mental health were also excluded (23–26). However, the first author (YD) and principal author (KT) did hand search the reference list of the reviews for studies relevant to ASC populations to ensure that all empirical studies were included in the final analysis for the review.

### Study Selection and Data Extraction

**Figure 1:**
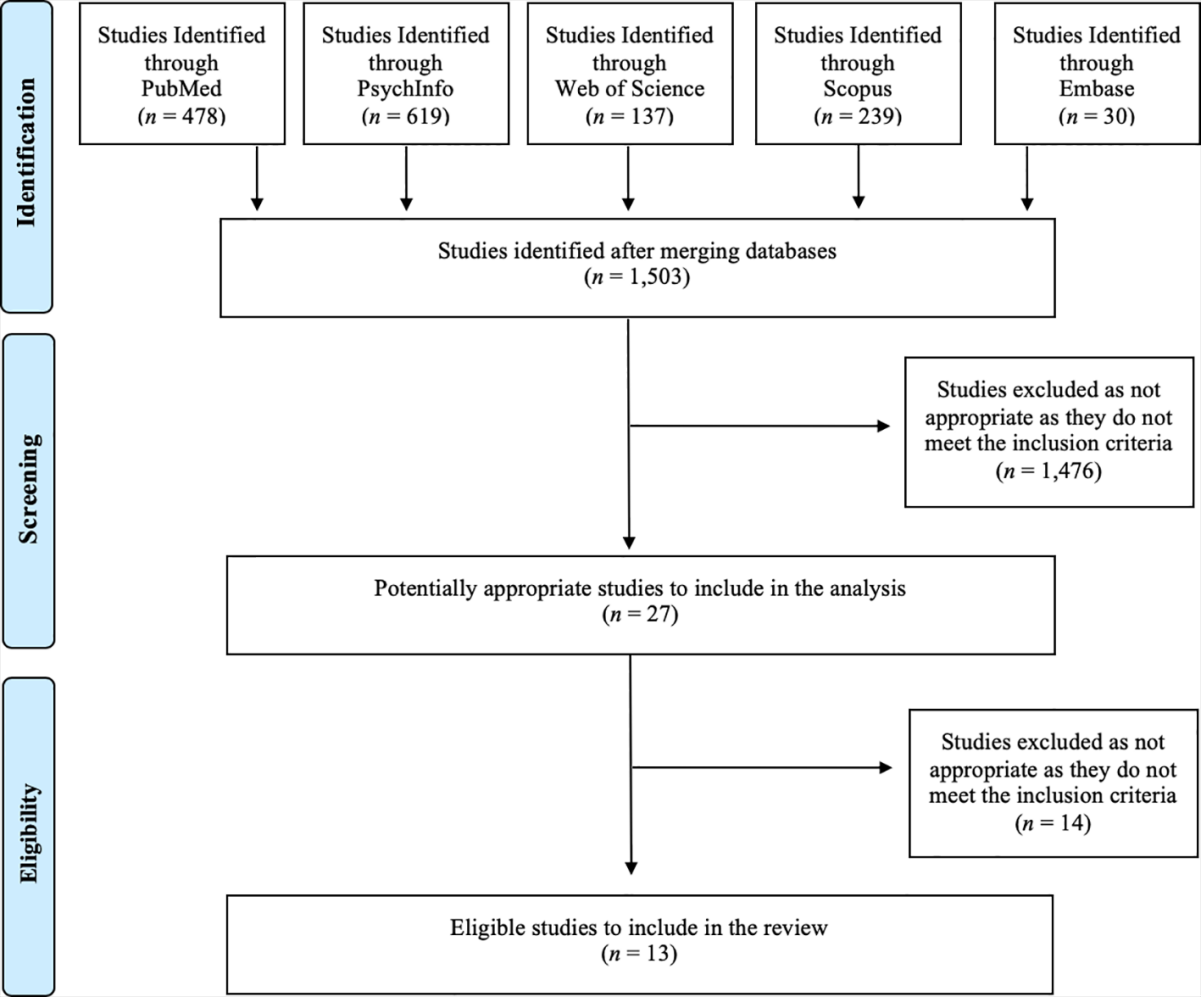
PRISMA flow diagram of the studies retrieved for the review.

The database searches and study selection were undertaken by five authors. **Figure 1** provides the PRISMA flow diagram (21) of the studies retrieved for the review. The PubMed yielded 478 publications, PsycINFO 619 publications, Web of Science 137 publications, Scopus 239 publications and Embase 30 publications. Together the five searches yielded 1,503 publications. Publication titles and abstracts were screened initially, and eligibility was established by reading the full texts. Duplicates and those that did not meet the inclusion criteria outlined below were removed. A manual reference search by exploring the reference list of the eligible publications was also conducted by the first author (YD) and principal author (KT) to identify additional papers of relevance. A total of 13 papers were then concluded as being relevant.

### Data Analysis

Due to the significant heterogeneity between participant characteristics and outcome measures used, it was not feasible to pool the data and undertake a meta-analysis. Therefore, a narrative method of analysis was undertaken.

## Results

### Study Selection

**Table 1** summarizes information about the studies included in the systematic review based on the inclusion criteria. Publications are stratified according to publication type and chronological order, giving insight into how research in this area has developed from case series to RCTs. This paper restricts itself to summarizing the findings narratively. CR interventions and ASC was examined in 13 studies; four RCTs (10, 18–20); two non-randomized control trials (27, 28); four case series (30–32, 34); two feasibility studies (29, 33) and one case study (35). **Table 1** includes a summary of limitations associated with each study that may impact interpretation.

**Table 1:** Characteristics of the included studies.

**RANDOMIZED CONTROL TRIALS**								
**Study**	Aim of the study	Design	Inclusion Criteria	Sample characteristics	Intervention	Outcome measures	Summary of results	**Limitations**
Bölte et al. (18)	Preliminary evaluation of the development of a computer-based program (using social cognition remediation) to test and to treat facial affect in autistic people.	RCT	Participants to have a diagnosis of autism or Asperger’s*.	*n* = 10 autistic people aged 16–40.	Computer-based program to test using social cognition remediation.	International Affective Picture System* and fMRI-scans.	The results indicated a statistically significant improvement in facial affect in the intervention group post the computer-based program.	Small sample size affected power and limited the generalizability of findings. Gender bias by only including male participants.
De Vries et al. (10)	To investigate the effect of a working memory flexibility-training compared to an active control condition.	RCT	Prior diagnosis of ASC, IQ ≥80* and absence of a seizure disorder.	*n* = 121 children. Diagnosed with ASC.	‘Braingame Brian’: executive function training. 25 Group sessions.	Corsi-BTT, BRIEF, SSRT, N-Back task, Gender-emotion switch-task*	The trend toward improvement in working memory and cognitive flexibility. Not feasible for autistic children.	High attrition rate. Targeted isolated aspects of cognition with limited functional impact. Does not specify participants gender
Miyajima et al. (20)	To explore the effects of CRT using the frontal/executive program for ASC compared to treatment as usual (normal supportive psychotherapy).	RCT	Outpatients younger than aged 60 & ≥9 years of formal education.	*n* = 14 adult outpatients. Diagnosed with ASC.	CRT using the frontal/executive program—44 individual sessions. (2 sessions a week).	BACS-J, WCST, CPT, ScoRS-J, LASMI*	Intervention group improved in working memory, executive functioning, verbal fluency and planning.	Small sample size. No follow-up investigation to determine the persistence of the effect.
Eack et al. (19)	To examine the efficacy of CET for improving core cognitive (neurocognitive & social-cognitive) and employment outcomes in autistic adults.	RCT	Diagnosis of ASC, IQ ≥80*, adults aged 16-45 and young people aged 16–17.	*n* ***=*** 33 adult, 7 adolescent male outpatients. Diagnosed with ASC.	CET—60 h over 18 months. Individual and group sessions.	MCCB, WCST, MSCEIT, PERT, PEDT, PEAT, SCS*	CET significantly increased neurocognitive function & social cognition in comparison to the control group.	Small sample size affected power. Treatment conditions were not matched (hours of treatment).
**NON-RANDOMIZED CONTROL TRIAL**								
**Study**	**Aim of the study**	**Design**	**Inclusion criteria**	**Sample characteristics**	**Intervention**	**Outcome measures**	**Summary of results**	**Limitations**
Golan et al. (27)	To teach autistic adults to recognize complex emotions using interactive multimedia. Using social cognition remediation (SCR) to improve the theory of mind.	Non-randomized controlled	Diagnosis of ASC and not taking part in any other intervention.	*n* = 39 autistic adults (32 males and 7 females). 13 people in each condition.	SCR using the mind reading intervention. Individual and group sessions.	CAM, RME, RMV & RMF*	Significant improvements on measures of face and voice recognition following intervention.	Sample was not randomized. The use of computer-based tasks is different to real life.
Turner-Brown et al. (28)	To evaluate the efficacy of social cognition remediation to improve social-cognitive functioning in high functioning autistic adults.	Non-randomized controlled	Aged 18–55, ASC diagnosis, IQ ≥80.	*n* ***=*** 11 high functioning autistic adults	Group based Social Cognition and Interaction Training modified for autism.	FEIT, Hinting Task, SCSQ and SSPA*	Intervention group showed significant improvement in theory of mind & social communication skills.	Small sample size and the quasi-experimental nature of the design where the sample was not randomized.
**CASE SERIES**								
**Study**	**Aim of the study**	**Design**	**Inclusion criteria**	**Sample characteristics**	**Intervention**	**Outcome measures**	**Summary of results**	**Limitations**
Eack et al. (29)	To examine the feasibility and potential efficacy of CET in autistic adults.	Feasibility study	ASC diagnosis, cognitive and social disability, IQ ≥80.	*n* = 14 young autistic adults (12 males 2 females).	CET. 60 hours over 18 months. Individual and group sessions.	CSQ-8, NIMH MATRICS, WCST, CSSCEI, PERT, SCP*	Improvements in cognitive difficulties and social cognition.	Small sample size, limiting generalizability of the results. Gender bias.
Hajri et al. (30)	To investigate whether CRT improves cognitive function in autistic children.	Cross-sectional	ASC diagnosis, cognitive difficulties*, on stable dose/type of medication.	*n* = 16 autistic children, aged 6-21 with regular school curriculum.	Individual CRT 22 sessions (one session per week).	CPM, VSFT, PFT, SF, DST, CARS*	CRT showed significant positive effects on neurocognition.	Small *N*. No follow-up investigation to determine the long-term persistence of the effect. Does not specify participants gender.
Tchanturia et al. (31)	To examine the treatment response of group CRT in anorexia nervosa patients with high or low autistic traits.	Cross-sectional	Adults with a diagnosis of anorexia nervosa.	*n* = 35 adult inpatients with AN diagnosis.	Group CRT 6 once-weekly sessions.	Motivational ruler, DFlex, patient feedback questionnaire*	No improvements following CRT for patients with high autistic traits.	23% of participants were only assessed for ASC with self-report questionnaires.
Hajri et al. (32)	To evaluate CRT’s effectiveness for autistic children on executive functions, clinical symptoms & education.	Cross-sectional	ASC diagnosis, cognitive difficulties*, on stable dose/type of medication.	*n* = 16 autistic children and adolescents, aged 6–21 with regular school curriculum.	Individual CRT adapted for young autistic people, once weekly.	CPM, HSCT, ROCF, CARS	Significant improvement in intellectual abilities, executive functions and clinical symptoms following CRT	Small sample size. No control group. Does not specify participants gender.
Okuda et al. (33)	To investigate the effectiveness and feasibility of CRT for ASC individuals.	Single-group pilot study	ASC diagnosis, aged 18–50, IQ ≥80.	*n* =16 female outpatients (4 adolescents and 12 adults).	Individual CRT, 10 sessions (weekly or biweekly).	Brixton, TMT, ST, WCST, ROCF, CFS*	Increase in patient’s central coherence following CRT, but not statistically significant.	Small sample size which results in a lack of power to detect statistical differences.
Dandil et al. (34)	To examine the difference in the effects of individual CRT for patients with AN and high autistic features (HAF).	Cross-sectional	Adult females with a diagnosis of AN and completed the AQ-10*.	*n* = 99 adult female inpatients diagnosed AN. (59 with HAF).	Individual CRT, 10 sessions (weekly or biweekly).	ROCF, Brixton SAT, DFlex*	HAF patients showed improvement in cognitive flexibility but not central coherence.	Participants were only assessed using ASC screening tools and not the full ASC diagnostic measures.
**SINGLE CASE STUDY**								
**Study**	**Aim of the study**	**Design**	**Inclusion criteria**	**Sample characteristics**	**Intervention**	**Outcome measures**	**Summary of results**	**Limitations**
Dandil et al. (35)	To investigate the feasibility of individual CRT for a complex single case study of anorexia nervosa (AN) and ASC.	Case study	Diagnosis of ASC and anorexia nervosa.	*n* = 1 inpatient autistic female aged 21 diagnosed with AN.	Individual CRT 13 sessions (twice a week).	DFlex, ROCF, Motivational ruler, Brixton SAT *	CRT indicated improvements in cognitive flexibility and central coherence.	Single case study, therefore, hard to generalise to a larger population.

*Diagnosis of ASC = (1, 36, 37), IQ, Intelligence Quotient (38); Cognitive difficulties, The Cognitive Styles and Social Cognition Eligibility Interview (39); AQ-10, The 10-Item Autism Spectrum Quotient (40); Corsi-BTT, The Corsi block tapping task (41); BRIEF, Behavioral Rating Inventory of Executive Functioning (42); SSRT, The stop-signal reaction time (43); N-back task (44, 45), Gender-emotion switch-task (46). BACS-J, The Brief Assessment of Cognition in Schizophrenia- Japanese version (47); CPT, Continuous Performance Test (48); SCoRS-J, Schizophrenia Cognition Rating Scale-Japanese version (48); LASMI, Life Assessment Scale for the Mentally Ill (49); MCCB, MATRICS Consensus Cognitive Battery (50); MSCEIT, The Mayer–Salovey–Caruso Emotional Intelligence Test (51); CAM, Cambridge Mindreading Face-Voice Battery (52); RME, Reading the Mind in the Eyes (53); RMV, Reading the Mind in the Voice task (54); RMF, Reading the mind in the films (55); FEIT, The Face Emotion Identification Test (56); The Hinting Task (57); SCSQ, Social Communication Skills Questionnaire (58); SSPA, Social Skills Performance Assessment (59); CSQ-8, Client Satisfaction Questionnaire-8 (60); NIMH MATRICS, Consensus Cognitive Battery (50); WCST, Wisconsin Card Sorting Test (61); CSSCEI, Cognitive Style and Social Cognition Eligibility Interview (39); PERT, Penn Emotion Recognition Test-40 (62); PEDT, Penn Emotion Discrimination Task (63); PEAT, Penn Emotional Acuity Test (64); SCS, the Social Cognition Profile (39); CPM, Raven Progressive Matrices (CPM); (65); VSFT, Verbal and Semantic Fluency Tests (66); PFT, phonemic fluency task (67); SF, Semantic Fluency (68); DST, Digit-span task (69); CARS, Childhood autism rating scale (70, 71); HSCT, Hayling Sentence Completion Task (72); TMT, Trail making test (73); ST, Stroop Test (74); CFS, Cognitive Flexibility Scale (75); DFlex, The Detail and Flexibility Questionnaire (76); ROCF, The Rey–Osterrieth Complex Figure (77); Brixton SAT, The Brixton Spatial Anticipation Test (72).

### Findings

#### Interventions

All of the interventions used in these studies were originally developed for use in other populations, rather than specifically designed for autistic people. The majority of the studies (*n* = 7) identified in the systematic review evaluated Cognitive Remediation Therapy (CRT) interventions, originally designed for use in people with schizophrenia (78). Three studies utilized this original program, which included modules targeting cognitive flexibility, memory, and planning (20, 30, 32). More recently CRT has been adapted for use in people with AN, a manualized approach focusing on cognitive flexibility and additions were made to address central coherence—meaning that the adapted manual for eating disorders added cognitive exercises supporting participants in “bigger picture” thinking (11). This intervention was used in four studies (31, 33–35).

Two studies investigated Cognitive Enhancement Therapy (19, 29). CET is a manualized CR approach originally developed for people with schizophrenia that integrates computer-based neurocognitive training with group-based training in social cognition (39). Other interventions focusing on social cognition remediation include the Social Cognition and Interaction Training (SCIT); (79), which is a group intervention that was initially developed for adults with psychotic disorders. One study used SCIT modified for autism (28). SCIT targets various components of social cognition, including theory of mind, emotion recognition, and attributions as well as social interaction skills.

The use of social cognition remediation interventions using computer-based protocols to improve the theory of mind in autistic people was also used in a further two studies (18, 27). This included the Mind Reading intervention (80) which is a computer-based program on emotions and mental states. It is aimed for children and adults to improve their ability to recognize emotions in others. The three main sections in the program include the Emotions Library (allows people to browse through different emotions and emotion groups), the Learning Centre (utilizes lessons and quizzes to teach emotions in a structured and directive way) and the Games Zone (comprises of educational games while studying about emotions). Finally, one study (10) utilized the cognitive flexibility and working memory modules of a computerized executive functioning training program designed for children with Attention Deficit Hyperactivity Disorder (ADHD) known as ‘Braingame Brian’ (81).

#### Randomized Control Trials

The first RCT study on the social cognition remediation effects on ASC provided a preliminary evaluation of the use of social cognition remediation, using a computer-based program they designed to test and to teach the recognition of facial affect and improve theory of mind in autistic adults (18). Five of the participants were randomly assigned to receive the computer treatment and five were in the control group (*n* = 10). The results indicated a statistically significant improvement in facial affect (*p <*0.05, *d* = 3.59) in the intervention group post the computer-based program.

Cognitive remediation effects on ASC were then explored through working memory and cognitive flexibility-training for autistic children using computerized cognitive training (10). The final sample included 121 children who were randomly assigned to an adaptive cognitive flexibility training, an adaptive working memory training or non-adaptive training. ‘Braingame Brian’ (81) a computerized executive function training with game elements was used. They reported no significant change in neurocognitive and psychosocial outcomes and disorder-related symptoms (*p* >.05) with a small effect size (η_p_^2^ = 0.01–\0.06). A marginal effect of improvement in working memory, cognitive flexibility, and in disorder-related symptoms was also highlighted.

The following RCT investigated the effects of CRT using the frontal/executive program (FEP) for ASC compared to treatment as usual (normal supportive psychotherapy), (20). The final sample of 14 autistic adults were randomly assigned to one of the groups. Of the initial 15 adults, seven (three males and four females) were assigned to the intervention group, seven (five males and two females) were assigned to the control group and one person dropped out. After completion in the FEP program they reported the intervention group significantly improved in cognitive functioning (*p* = 0.018) working memory (*p* = 0.018), verbal fluency *(p* = 0.008) and planning (*p* = 0.012) with a large effect size (*d* = 2.73).

To date, the final RCT then examined the efficacy of Cognitive enhancement therapy (CET); (39) in an 18-month CR intervention for improving the core cognitive and employment outcomes in 54 autistic adults (19). Participants were randomly allocated to either the CET intervention which incorporates computerized neurocognitive and social-cognitive remediation or an active Enriched Supportive Therapy (EST); (82) comparison focused on psychoeducation and condition management. Participants included 32 adults and seven adolescents. The results indicated that CET significantly (*p* <.05) increased neurocognitive function in comparison to EST (*p* = 0.13) and there was a medium effect size (*d* = 0.46). Although EST was linked to social cognitive improvements, CET demonstrated a larger improvement in social cognition (*p* = .020) with a medium effect size (*d* = 0.58).

#### Non-Randomized Control Trials

The first non-randomized control trial utilized social cognition remediation interventions, which concentrated on improving the theory of mind in autistic adults (27). This systematic review will focus on experiment two they reported in their study where the outcome focused on social cognition. The authors used the Mind Reading intervention (80) for autistic adults with weekly support of a tutor for the intervention group. This group was then matched to a control group of autistic adults attending social skills training then another general population control group. 13 adults were included in each group (*n* = 39). Improvement of emotion recognition skills was the target outcome measure. Participants were included through two support organizations and two colleges for autistic people. Autistic participants had all been diagnosed using the established criteria (83). Self-reported autistic traits were then assessed using the Autism Quotient (AQ); (84) and the parent version of the AQ (85). The authors found significant improvements on measures of face and voice recognition in the intervention group (*p <*0.001, *d* = 0.14).

A subsequent non-randomized control trial then aimed to evaluate the efficacy of social cognition remediation to improve social-cognitive functioning in high functioning autistic adults (28). The authors investigated the impact of group-based Social Cognition and Interaction Training (SCIT); (79) modified for autism to treatment as usual in a pilot study using a non-randomized control trial. The sample included 11 participants. Six adults received SCIT for autism and five received treatment as usual. They reported that participants who received the intervention showed significant improvement in theory of mind skills and trend level improvement in social communication skills (*p <*0.05). The within-group effect size also indicated a large treatment effect (*d* = 0.94).

#### Case Series and Case Study

Two different types of case series were identified: studies focusing on pre and post CR assessments in single or multiple case series and feasibility studies. The first case series in this area was a feasibility study (29), in which authors examined the feasibility of Cognitive enhancement therapy (CET); (82) for autistic adults. Participants included (*n* = 14) young adults (12 males and two females). They were recruited to participate in an uncontrolled, 18-month trial of CET adapted for autistic adults. The authors reported that CET made a statistically significant (*p <*0.001) improvement in cognitive performance, social behavior and social cognition and social functioning proved to be the largest domains of improvement within this study with a large effect size (*d* = 1.40–2.29).

A cross-sectional study then investigated whether a CRT intervention developed for adults with schizophrenia (78) improved cognitive function in autistic children (30). 16 children referred to the children and adolescent Psychiatric Department in Tunisia completed the modified version of CRT adapted for children. The main outcome measures; clinical symptoms, executive function and school performance was calculated at baseline and one week after completing the CRT intervention. CRT showed significant positive effects on clinical symptoms (*p* = 0.001) and working memory (*p* = 0.006) for autistic children.

The treatment response of group CRT for people with AN (11) was then examined in patients with AN and high or low autistic traits (31). Participants included 35 adults with a diagnosis of AN by a consultant psychiatrist based on the Diagnostic and statistical manual of mental disorders, fifth edition (DSM-IV); (1) with high or low autistic traits (40) on an inpatient eating disorder unit. All participants completed self-report questionnaires on motivation and thinking styles before CRT and then after CRT. The results indicated that patients with high autistic traits showed no statistically significant (*p* >.05) improvements and negligible effect sizes in the self-reported outcome measures for CRT (*d* = 0.10).

In another cross-sectional study, CRT (78) adapted for autistic children and adolescents was used to evaluate the effectiveness on executive functions, clinical symptoms and school performance (32). The final sample included 16 young people. Participants were evaluated pre-CRT intervention and post-CRT intervention. The results supported that after CRT, the young autistic people showed significant improvements in intellectual abilities, phonemic flexibility, working memory, school results and clinical symptoms (*p <*0.05) with a small effect size (r^2^ = 0.95).

Two more recent case series have examined the use of a CRT intervention for people with AN in both autistic people, and people with high autistic traits (11). A small prospective pilot study in Japan (33) then aimed to investigate the effectiveness and feasibility of CRT for anorexia nervosa (11) for ASC individuals. 19 patients diagnosed with ASC were included in the study. Outcome measures were given to participants pre-CRT, post-CRT and at 3-month follow-up. Results demonstrated an increase in the patient’s central coherence following CRT. However, these results were not statistically significant (*p* >.05) although the magnitude of the effect was large (*d* = 0.80).

Furthermore, a preliminary study aimed to examine the difference in the effects of individual CRT treatment for adult female’s diagnosed with anorexia nervosa with either high or low ASC characteristics (34). In total 99 inpatients on an eating disorder unit were initially included in the intervention. Out of the 99 inpatients, 59 patients met the threshold for high autistic traits using the 10-Item Autism Spectrum Quotient (AQ‐10); (40). However, there was only complete data for 25 (41%) of the patients that scored with high autistic features. The results indicate that participants with high autistic features showed significant improvements in cognitive flexibility (*p <*0.001) after CRT with a large effect size (*d* = 0.77). However, scores did not show a statistically significant improvement in central coherence (*p* >.05), although there was a larger mean difference of −0.15 (0.38).

The clinical implementation of CRT for AN in ASC populations was investigated in more detail in a single case study (35). To the authors’ knowledge, this is the only case study in this area. They investigated the feasibility of individual CRT adapted for AN (11) for a complex single case study of a 21-year-old inpatient female diagnosed with anorexia nervosa and ASC. The paper reflects on possible adaptations to enhance the efficacy of CRT for this population. The results suggested improvements in central coherence and cognitive flexibility following CRT. The authors argue that this study provides preliminary support of the efficacy of individual CRT, before proceeding to more complex psychological work, such as cognitive behavioral therapy.

## Discussion

### Summary of Main Findings

This systematic review aimed to contribute to the field of ASC by presenting a narrative synthesis of all studies of CR interventions and ASC. Thirteen studies met the pre-determined inclusion criteria. However, several methodological challenges made it difficult to appraise the empirical studies comprehensively. Overall, the results suggest CR interventions are potentially effective in improving social cognition and cognitive functioning in ASC. The review findings are broadly consistent with an earlier review that focused on the current status of CR for psychiatric disorders (26). However, the review only included two RCTs on CR interventions and ASC (10, 20).

The four RCTs presented in this paper, although varying in terms of study design, aims and sample characteristic, depict how CR interventions are currently being implemented in diverse settings for ASC. The first RCT study on the social cognition remediation effects on ASC (18) found an improvement in social functioning and theory of mind in autistic people. Though, limitations of this study included its small sample size, which affected power and limited the generalizability of the findings and its gender bias by only including male participants. Two further studies (10, 20) then supported the efficacy of CR interventions in improving working memory. One of these studies reported that a CRT intervention using the frontal/executive program is effective in improving executive functioning in ASC patients, and in particular to those with impaired frontal lobe function (20). However, the limitations of this RCT included its small sample size. There was also no follow-up investigation to determine the persistence of effect. A following RCT then found that CET increased neurocognition function and improved non-social and social cognition for ASC individuals in comparison to the control group (19). However, the small sample size affected power and the treatment conditions were also not matched in terms of the hours of treatment received.

Despite the limitations of the RCTs, three of the studies (18–20) support the feasibility and potentially effective treatment of CR interventions for core cognitive and social cognition domains in autistic adults. However, one of the RCTs found that the computerized executive training with game elements ‘Braingame Brian’ (81) at present will not be feasible for autistic children due to the absence of a clear effect and high attrition rate (10).

The two non-randomized control studies included in this review utilized computer-based social cognition remediation to improve the theory of mind in autistic people (27, 28). Both their results showed an improvement in social cognition for autistic people following the interventions. However, it is important to note that while there is an increasing trend in the use of computer-based interventions and approaches, at present there is no research evidence supporting that they are more effective than non-computer-based interventions. Although computer-based interventions can provide controlled and structured assessments, they can be different to real-life. For instance, in one of the studies, participants reported that they found it easier to recognize emotions and mental states on the computer instead of in real-life social situations (27). Hence, the interpretation of findings from computer-based programs to real-life functioning should be interpreted with caution.

Studies identified were predominantly case series and have supported the feasibility and acceptability of individual CR interventions with positive feedback from participants (29, 30, 32–34). However, the case series identified had small sample sizes which resulted in a lack of power to detect statistical differences and limits the generalizability of the findings. The design of these studies also cannot draw causal conclusions about the effect of CR interventions. Additionally, one cross-sectional study examined group CRT for those with high autistic traits and found no improvements following the intervention (31). The intervention used was CRT for anorexia nervosa (11) that may have needed to be adapted further for ASC participants. In particular, the group environment may have presented additional difficulties for participants on the spectrum.

Notwithstanding the findings from the studies included in the review, caution is apt in concluding the current evidence for several other reasons. Some participants in the studies on AN populations did not have a full ASC diagnosis and were only assessed using ASC screening tools and self-report questionnaires (31, 34). However, people with AN on the spectrum are often undiagnosed, and these screening tools are used clinically to inform diagnostic referrals (86).

Furthermore, the studies in this review used various CR programs for a diverse range of age groups, with a variety in intensity, duration, measurement tools and target of the cognitive domain. Thus, this diversity may contribute to inconsistent findings and effect sizes as evident in **Table 1**. In addition, the potential role of other variables is unclear from the present research. For example, the training each facilitator may have had for delivering CR interventions may have also differed, and the results may have been affected by participants learning effect. Finally, ASC research typically focuses on males which are also evident in some of the studies included in this review. Compared to males, females are at elevated risk of their ASC going undiagnosed as their difficulties are frequently mislabeled or missed entirely (87). This may be a result of the “female phenotype” of ASC characteristics, with females showing fewer repetitive and restricted behaviors than males and prone to “camouflage” their social difficulties (6, 88). This review suggests that the field of eating disorders is leading on the inclusion of females with autistic traits in this field of research, with the highest population of female participants self-reporting autistic traits in this review included from this population (31, 34).

It is notable that all of the interventions identified in this systematic review were originally developed for use in other clinical populations (brain injury, schizophrenia, AN, ADHD). Previous research on psychological interventions suggests that they may need to be adapted for ASC populations (89). While larger studies are needed, the single case study presented in this review explored adapting CRT on an individual level, highlighting potential adaptations that can be useful for the ASC population (35). There is a no ‘one-size-fits-all’ approach as each autistic person is unique, however, possible modifications to CR interventions can include clarifying sensory sensitives, accommodating certain routines and rituals, providing clear examples using a didactic style, reducing the number of skills taught and checking understanding. Other adaptations included the individuals wider social network and using colorful visual materials. One of the case series included in this review also made adaptations to CRT for young autistic people by making the tasks simpler and “funnier” (32). Furthermore, the CET intervention was also adapted, by including psychoeducation about ASC, providing greater support with the interventions, and adapting the computer program to eliminate sounds that might be uncomfortable for participants with sensory difficulties (29).

Although some of the studies were limited, they are informative and have useful implications when considering future developments in clinical practice and service provision. Early intervention program for ASC are increasingly being held to the matching standards as traditional medical trials. Thus, the practical and collaborative nature of CR interventions can be appealing for the ASC population as an early intervention. The findings also indicate that clinicians delivering CRT interventions for conditions, particularly eating disorders, should be mindful of the possibility of patients presenting with ASC, or high autistic traits, as this may impact outcomes. Collecting information on ASC characteristics such as social and communication difficulties, restricted and repetitive behaviors, and differences in neuropsychological functioning can assist clinicians in making suitable modifications to meet individual needs.

Implications for research include further rigorous studies with large sample sizes such as RCTs that help to better understand the mediating and moderating factors for CR interventions in ASC, as well as the effectiveness and acceptability of CR interventions. Moreover, it is important to increase research into establishing reliable and valid outcome measures through developing measures tailored to the specific needs of individuals with ASC and determining normative thresholds for this population.

### Limitations

This systematic review has several limitations that should be noted. First, although we undertook the search using several databases, we excluded non-English language publications. Second, while we searched the published literature systematically, the potential publication bias could not be eliminated, for instance, retrospectively searching trial registers for any potentially unpublished studies. Finally, the various interventions and outcome measures used in the identified studies prevented a meta-analysis, limiting our ability to draw conclusions around efficacy or to assess the risk of bias.

### Conclusions

To our knowledge, this is the first paper that presents a narrative synthesis of all studies of CR interventions in ASC, and the first paper delineating its research development from single case studies to RCTs. The review findings indicate that CR interventions are potentially effective in improving social cognition and cognitive functioning in ASC. However, the generalizability of the included empirical studies was hampered by several methodological limitations. To further strengthen our understanding of the effectiveness of CR interventions for ASC, future studies should focus on [1] developing the evidence base about the mediating and moderating mechanisms of CR interventions in ASC, [2] exploring the long-term effects of the treatment, [3] integrating treatment approaches that target both non-social and social cognition difficulties, [4] devising further large scale RCTs exploring the effectiveness of CR interventions in both adolescent and adult populations using age-appropriate valid and reliable outcome measures, and taking into account the heterogeneity in neuropsychological functioning in ASC.

## Data Availability Statement

The original contributions presented in the study are included in the article/supplementary material; further inquiries can be directed to the corresponding author.

## Author Contributions

All authors contributed to the literature search (YD, KS, EK, CT, and KT). KT was the principal investigator and supervisor of the study. YD drafted the manuscript, interpreted the main findings, assembled and analyzed the data, and wrote the first paper. All authors contributed to the article and approved the submitted version.

## Funding

This work was supported by the Health Foundation, (Ref: AIMS ID: 1115447) and by a grant from the Maudsley Charity.

## Conflict of Interest

The authors declare that the research was conducted in the absence of any commercial or financial relationships that could be construed as a potential conflict of interest.
